# Metagenomic analysis of viral nucleic acid extraction methods in respiratory clinical samples

**DOI:** 10.1186/s12864-018-5152-5

**Published:** 2018-10-25

**Authors:** Dan Zhang, Xiuyu Lou, Hao Yan, Junhang Pan, Haiyan Mao, Hongfeng Tang, Yan Shu, Yun Zhao, Lei Liu, Junping Li, Jiang Chen, Yanjun Zhang, Xuejun Ma

**Affiliations:** 1 Key Laboratory for Medical Virology, National Health and Family Planning Commission, National Institute for Viral Disease Control and Prevention, Chinese Center for Disease Control and Prevention, Changping District, Beijing, 102206 China; 2 Institute of Microbiology, Zhejiang Provincial Center for Disease Control and Prevention, Hangzhou, 310051 China; 3 Department of Pathology, Children’s Hospital, Zhejiang University School of Medicine, Hangzhou, 310013 China; 4 College Of Medical Technology, Zhejiang Chinese Medical University, Hangzhou, 310013 China

**Keywords:** Illumina Hiseq, Viral nucleic acid extraction methods, Respiratory clinical samples, Metagenomic analysis

## Abstract

**Background:**

Numerous protocols for viral enrichment and genome amplification have been created. However, the direct identification of viral genomes from clinical specimens using next-generation sequencing (NGS) still has its challenges. As a selected viral nucleic acid extraction method may determine the sensitivity and reliability of NGS, it is still valuable to evaluate the extraction efficiency of different extraction kits using clinical specimens directly.

**Results:**

In this study, we performed qRT-PCR and viral metagenomic analysis of the extraction efficiency of four commonly used Qiagen extraction kits: QIAamp Viral RNA Mini Kit (VRMK), QIAamp MinElute Virus Spin Kit (MVSK), RNeasy Mini Kit (RMK), and RNeasy Plus Micro Kit (RPMK), using a mixed respiratory clinical sample without any pre-treatment. This sample contained an adenovirus (ADV), influenza virus A (Flu A), human parainfluenza virus 3 (PIV3), human coronavirus OC43 (OC43), and human metapneumovirus (HMPV). The quantity and quality of the viral extracts were significantly different among these kits. The highest threshold cycle(Ct)values for ADV and OC43 were obtained by using the RPMK. The MVSK had the lowest Ct values for ADV and PIV3. The RMK revealed the lowest detectability for HMPV and PIV3. The most effective rate of NGS data at 67.47% was observed with the RPMK. The other three kits ranged between 12.1–26.79% effectiveness rates for the NGS data. Most importantly, compared to the other three kits the highest proportion of non-host reads was obtained by the RPMK. The MVSK performed best with the lowest Ct value of 20.5 in the extraction of ADV, while the RMK revealed the best extraction efficiency by NGS analysis.

**Conclusions:**

The evaluation of viral nucleic acid extraction efficiency is different between NGS and qRT-PCR analysis. The RPMK was most applicable for the metagenomic analysis of viral RNA and enabled more sensitive identification of the RNA virus genome in respiratory clinical samples. In addition, viral RNA extraction kits were also applicable for metagenomic analysis of the DNA virus. Our results highlighted the importance of nucleic acid extraction kit selection, which has a major impact on the yield and number of viral reads by NGS analysis. Therefore, the choice of extraction method for a given viral pathogen needs to be carefully considered.

**Electronic supplementary material:**

The online version of this article (10.1186/s12864-018-5152-5) contains supplementary material, which is available to authorized users.

## Background

Next-generation sequencing (NGS) is an attractive approach to diagnosis of infection and might serve as a great potential method to identify viruses, bacteria, and fungi from a range of biological and environmental samples in clinical diagnostic and reference labs [1, 2]. Various NGS approaches provide solutions for detection of purified and concentrated viruses from culture, however, direct identification of viral genomes from clinical specimens using NGS methods still has its challenges, including noise from the host or microbiota cells and the limited viral RNA and DNA quantities [3, 4].

Numerous protocols for viral enrichment and genome amplification have been described in literature [5–7]. Commonly employed protocols such as sample filtration [8], nuclease digestion, ultracentrifugation, and random pre-amplification of RNA or DNA in separate reactions would be particularly useful for increasing the signal-to-noise ratio in the viral analysis of biological samples in which the levels of nucleic acid background are high. In principle, these protocols can significantly reduce the proportion of human and bacteria reads and increase the number of viral reads. In fact, filtration and nuclease treatment slightly decreased the number of virus reads and the number of viruses identified [6]. Pre-amplification using random RT-PCR resulted in detection of fewer viruses, more overlapping sequences, but lower genome coverage [6]. Amplicon-based NGS only detected pre-defined targets, thus possibly missing viruses or novel virus strains. Apart from these methods, a crucial step in the molecular detection of viruses in clinical specimens is the efficient extraction of viral nucleic acids [7]. Higher virus-related yields of the extracts meant better sensitivity in the subsequent detection analysis. Thus the extraction method selected may determine the sensitivity and reliability of diagnostic NGS [9]. As sample types greatly influence the composition of a sequencing read due to the complexity of clinical materials, it is therefore valuable to use the nucleic acid extracted directly from clinical specimens without enrichment to evaluate extraction efficiency.

In this study, we assessed the viral nucleic acid extraction efficiency of four commonly used Qiagen kits: QIAamp Viral RNA Mini Kit (VRMK), QIAamp MinElute Virus Spin Kit (MVSK), RNeasy Mini Kit (RMK), and RNeasy Plus Micro Kit (RPMK). Among them, the VRMK [10–13], MVSK [14–16] and RMK [3, 17, 18] were described in the literature on NGS-based detection using respiratory specimens. The performance of these kits for viral nucleic acid extraction was compared with regard to simultaneous isolation of viral DNA and RNA by qRT-PCR analysis. The number of reads containing five different viruses, distribution of viral sequencing reads into taxonomic categories, and the percentage of virus-specific reads generated by sequencing on the Illumina Hiseq 2500 system were evaluated in parallel using identical NGS processes and bioinformatics analyses. The mixed samples were from two nasopharyngeal aspirate specimens, which contained an adenovirus (ADV), influenza virus A (Flu A), human parainfluenza virus 3 (PIV3), human coronavirus OC43 (OC43), and human metapneumovirus (HMPV) without any pre-treatment approaches. These viruses were chosen to cover a wide range of different viral properties: non-enveloped DNA virus (ADV), enveloped single-stranded, segmented RNA virus (Flu A), enveloped negative-stranded RNA virus (PIV3, HMPV), and enveloped positive-stranded RNA virus (OC43).

## Results

### Comparison of extraction kit performance

For a fair comparison of four commercially available viral nucleic acid extraction kits, the same amounts of starting sample and elution buffer were used and five different viruses with different characteristics were chosen: Flu A, OC43, HPIV3, HMPV, and ADV. Following the nucleic acid extraction, virus elutes were initially quantified by qRT-PCR to determine the performance of each kit. Later, for each kit, two parallel libraries from two extracted aliquots of the same sample were individually generated using the same sequencing protocols and bioinformatics analyses. The parallel results showed good repeatability for each kit (see Additional file 1).

According to the qRT-PCR results (Table 1), the highest Ct values for ADV and OC43 were obtained by using the RPMK. For Flu A, PIV3, ADV, HMPV, and OC43, the lower Ct values were achieved with the VRMK, while the MVSK had the lowest Ct values for ADV and PIV3. The RMK revealed the lowest detectability for HMPV and PIV3. All five viruses showed detectable amounts of the viral nucleic acid in the respective samples, except for Flu A, which was undetected with the RPMK.

**Table 1:** Comparison of different extraction kits based on average Ct values by qRT-PCR (*n* = 2)

Kit	ADV(Ct)	Flu A(Ct)	PIV3(Ct)	OC43(Ct)	HMPV(Ct)
VRMK	24.2 ± 0.1	**31.7 ± 0.3**	17.6 ± 0.1	**30.1 ± 0.5**	**24.9 ± 0.8**
MVSK	**20.5 ± 0.2**	37.1 ± 0.5	**15.8 ± 0.2**	34.1 ± 0.6	27.7 ± 0.0
RPMK	34.5 ± 0.6	/	19.6 ± 0.7	38.8 ± 0.4	28.9 ± 0.9
RMK	26.9 ± 0.5	36.8 ± 1.1	24.7 ± 0.6	36.4 ± 1.1	36.3 ± 1.1

The best result is presented in bold

The nucleic acid concentrations of the DNA preparation were close to 1 ng/μL, which is the amount theoretically required for the use of the Next® Ultra™ DNA library Prep Kit, according to the manufacturer’s recommendations (Table 2). The highest DNA concentration on average was found in samples extracted by the MVSK (95.2 ng/μL), while extraction with the RPMK resulted in the lowest average concentration (0.10 ng/μL). Moderate DNA concentrations were observed with the VRMK (7.12 ng/μL) and RMK (2.47 ng/μL).

**Table 2:** Nucleic acid concentration of samples used with different kits, determined by Qubit (mean concentration, *n* = 2)

Kit	Average concentration(ng/μL)	
	RNA^a^	DNA^b^
VRMK	b/d ^c^	7.12
MVSK	b/d	95.20
PPMK	b/d	0.10
RMK	b/d	2.47

^a^ RNA concentration after preparation

^b^ DNA concentration after preparation

^c^ b/d, below Qubit detection limit

**Fig. 1:**
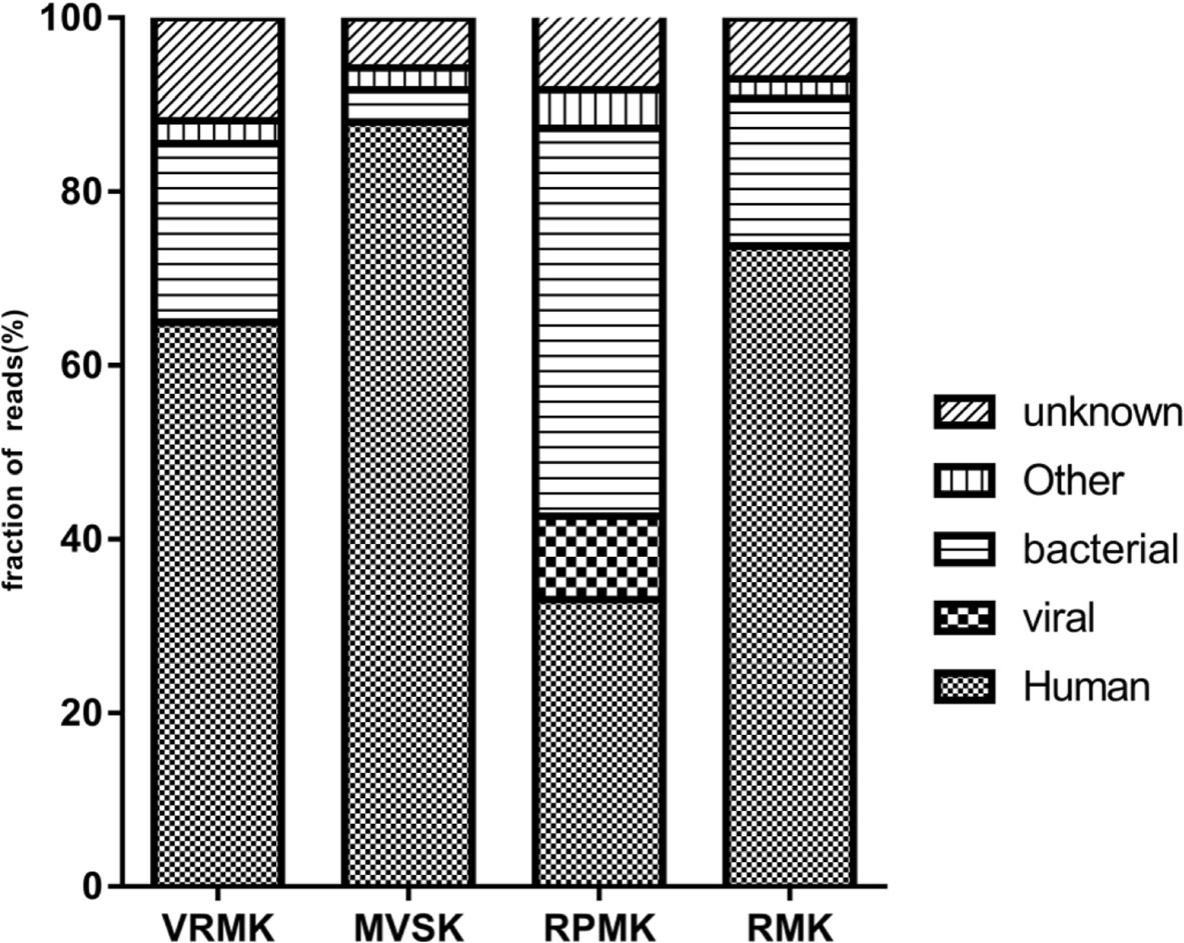
Metagenomic analysis of extraction efficiencies of four kits in clinical samples. Distribution of sequencing reads into taxonomic categories of viral, human, bacterial, other, and unknown origin

The Illumina sequencing of the respective libraries (*n* = 8) generated a total of 140 million paired-end reads, with a total of 21.08 Gbp of sequence information. On average, the percentage of bases with a quality score greater than 30 was 93.84% (Table 3). The amount of NGS data exhibited no particularly large differences among the eight samples. Reads passing quality filtering were mapped to the human reference genome hg18 using stringent criteria. The most effective rate of NGS data of 67.47% was observed with the RPMK (Table 3). The other three kits’ effectiveness rates of NGS data ranged between 12.1–26.79%. Most importantly, the highest proportion of non-host reads was obtained by the RPMK compared to the other three kits (Fig. 1).

**Table 3:** Comparison of quality control of average sequencing data for four kits

Kit	Raw data^a^(G)	Clean data^b^(G)	Remove host^c^(G)	Clean Q30^d^(%)	Effective Rate^e^(%)
VRMK	2.43	2.34	0.66	93.33	26.73
MVSK	2.77	2.73	0.32	95.18	12.10
RPMK	2.91	2.85	**1.88**	93.60	**67.47**
RMK	2.42	2.34	0.63	93.26	26.79

^a^Reads were not processed with bioinformatics analysis

^b^Reads were quality-filtered by removing low quality bases, reads shorter than 75 bp, and reads with low entropy

^c^Human reads were removed by aligning human (hg19) reference genome

^d^he percentage of bases with a quality score greater than 30

^e^Remove host /clean data

The best result is presented in bold

As shown in Table 4, extraction efficiencies of the four kits for five viruses were different. When aligned with the PIV3 reference genome sequence (GenBank accession number NC_001796.2), the RPMK generated sequences with up to a 100% breadth of coverage (94.5% nucleotide pairwise identity), with the highest PIV3 read number (58,338,663 reads), and the highest coverage for OC43 (0.83%) and HMPV (33.95%). The PIV3 full genome sequences were deposited into the GenBank under accession number MH411617. Compared to the RMK, the PIV3 genome reads were increased 5851-fold. In contrast, the lowest reads and coverage of ADV and Flu A were obtained by using the RPMK. The RMK produced the highest coverage percentage (79.32%) for the ADV. There were no considerable differences among the four kits in the read number and coverage for the Flu A (read 1–16, coverage 1.20–2.81).

**Table 4:** Comparison of the average read number and genome coverage of five respiratory viruses

Kit	Average sequencing reads (average genome coverage %)				
	ADV	Flu A	PIV3	OC43	HMPV
VRMK	13,391 (70.09)	**16 (2.81)**	149,002 (87.06)	1910 (0.23)	148 (14.11)
MVSK	9033 (56.50)	2 (2.45)	37,395 (75.32)	1555 (0.23)	/
RPMK	5992 (31.42)	2 (1.20)	**58,338,663 (100.00)**	**532 (0.83)**	**1130 (33.95)**
RMK	**17,215 (79.32)**	1 (1.80)	9970 (55.21)	1615 (0.25)	/

The best result is presented in bold

The proportions of sequence reads with significant hits for viruses, bacteria, unknown, other, and human entries are summarized in Fig. 1. We observed improvements in the rate of virus and bacteria detection in the clinical samples with the RPMK extraction. An average of 9.61, 44.67 and 33.02% reads were classified as known viruses, bacteria, and human, respectively. In contrast, only about 0.01–0.05% (average, 0.03%) of the valid sequences were classified as viruses with the other three kits.

## Discussion

Limiting factors in the nucleic acid extraction are lower pathogen concentration and specimen volume that could result in insufficient amounts of NGS starting material. Therefore, increasing sample volume is an effective solution for viral metagenomics analysis in order to improve the number of virus reads and genome coverage. In addition, increased sequencing capacity also improves the chances of virus detection [19]. Although the use of more massive throughput NGS platforms may increase the cost of use, it is still a reliable and effective method [20].

In this study, we compared the extraction efficiency of four commonly used Qiagen extraction kits (VRMK, MVSK, RPMK, and RMK) using the same amount of input and output (elute) of the same sample. In contrast to using cell-cultured reference viruses, this study focused on NGS-based detection and analysis of respiratory clinical specimens. We evaluated the performance of aforementioned four kits with NGS in terms of the ability of each kit to recover sequence information of five different DNA and RNA viruses in the clinical samples. To ensure the reliability and repeatability of experimental results, we set up the extraction (*n* = 8, 2 for each kit), qRT-PCR assay (*n* = 16, 2 for each extraction), and NGS analysis (n = 8, 2 for each kit) in duplicate.

With the RPMK procedure, samples are first lysed and homogenized in a highly denaturing guanidine-isothiocyanate-containing buffer, which immediately inactivates RNases to ensure isolation of intact RNA. The lysate is then passed through a gDNA eliminator spin column. This column, in combination with the optimized high-salt buffer, efficiently removes genomic DNA. Our results demonstrated that among all the tested kits, the RPMK performed best in the viral metagenomic analysis of the number of sequencing reads and genome coverage of the RNA viruses (PIV3, OC43, and HMPV), with Ct values ranging from 19.6 to 38.8, suggesting a broad adaptability of the RPMK extraction. The only exception occurred in the FluA, where only two viral reads were found. This phenomenon is not surprising because FluA was undetected by qRT-PCR after the RPMK extraction. However, no dramatic difference in the Flu A counts (1–16) or coverage (1.2–2.8) was found among all the tested kits due to the low viral concentration (Ct values 31–37) in the clinical sample. Additionally, the RPMK appeared not suitable for the ADV (DNA virus). Compared to other three kits, a relatively low numbers of the ADV sequencing reads (5992) and coverage (31.42%) were obtained using the RPMK. This might be attributed to the loss of viral DNA along with the removal of host genomic DNA after the RPMK extraction. Notably, the RPMK appeared to remove significant amounts of host genomic DNA. Compared to other three kits showing only small differences (12.1–26.79%, Table 3) in removing human reads, the RPMK effectively reduced the amount of human reads, dramatically increased the proportion of viral RNA (9.61%) and bacterial reads (44.67%) (Fig. 1), and even obtained the full PIV3 genome sequence, thus providing sufficient sequence information to confirm virus identity. With whole viral genome sequences, it can also inform likely phenotypes, including drug susceptibility or neutralization serotypes and may prove useful in viral transmission and evolution studies [21, 22]. To our best knowledge, this is the first study on viral metagenomics analysis of respiratory clinical samples without pre-treatment approach using the RPMK.

Interestingly, the ratio of viral reads and coverage obtained by the NGS was not well correlated with Ct values of the same sample, highlighting the importance and necessity of using different methods to evaluate extraction efficiency. The qRT-PCR results demonstrated that among four tested kits, the VRMK possessed highest extraction efficiency for the Flu A, OC43, and HMPV (lowest Ct value), and better performance than the RPMK in the extraction of PIV3 (Ct values 17.6 vs. 19.6). However, metagenomic analysis data showed that the RPMK exceeded other three kits in the obtained viral sequencing reads and/or genome coverage of the PIV3, OC43, and HMPV. Particularly in the case of PIV3, full length genome was obtained by the RPMK while only 87% genome coverage was achieved by the VRMK. Additionally, HMPV was detected by qRT-PCR with Ct values of 36.3 and 27.7 using the RMK and MVSK, respectively, but was missed by the NGS. As for the Flu A, a slight difference in the number of recovered NGS reads and coverage was observed (1–16 reads, 1.20–2.81% coverage), though the range of Ct values was 31.7–37.1. Our results illustrated that NGS-based detection should not be solely dependent on the Ct values of qRT-PCR, suggesting that the quality of the extracted nucleic acid are more crucial than the quantity and the quality of the viral extracts is significantly different among these kits.

With qRT-PCR analysis only, our data also showed that different kits may exhibit different extraction efficiency for the same and different viruses. Dramatic differences in the Ct values were observed among the five selected viruses (Table 1). Before pooling, the original Ct values for the ADV, Flu A, PIV3, OC43, and HMPV were 23.9, 26.5, 17.1, 29.2, and 27.1, respectively, which were lower than the results obtained using tested kits in this study. This may be explained by the use of automated extraction platform and the addition of carrier RNA in the original extraction methods, a typical way to increase the extract yield. The repeated freeze-thawing steps might also contribute to this bias. As appropriate kit choice can be a crucial factor in determination of experiment results, our results implied that no suitable kit is perfect for all the viral pathogens and the choice of extraction method for a given viral pathogen needs to be carefully considered.

Notably, viral RNA extraction kits are able to isolate viral DNA effectively. The VRMK, RPMK, and RMK are designed for viral RNA extraction, while the MVSK for both viral DNA and RNA extraction, according to manufacturer’s recommendations. Compared to the other thee kits, it is not surprising that with qRT-PCR analysis, the MVSK performed best with the lowest Ct value of 20.5 in the extraction of the ADV (DNA virus), while the VRMK, RPMK, and RMK achieved moderate and higher Ct values of 24.2, 34.5, and 26.9, respectively. Further NGS analysis results placed the extraction kits in the order of decreasing extraction efficiency as follows: RMK, VRMK, MVSK, and RPMK, indicating that the RMK is actually most suitable for the ADV identification, rather than the MVSK. We assumed that viral RNA extraction kits were also applicable for metagenomic analysis of the DNA virus, probably by effectively capturing the RNA transcripts of the DNA virus in the extracted clinical sample.

Our results provide contrasting evidence to a previous study [9], which reported the evaluation of four different commercial nucleic acid extraction kits with four different viruses and concluded that selection of kits has only a minor impact on the yield of viral reads and the read numbers obtained by NGS. The following factors might explain the differences between our study and previously reported research: 1) different extraction kits and tested viruses were used; 2) mixed aliquots from egg- or cell-cultured reference viruses were used in previous work, while mixed clinical samples were used in the current study; 3) each extracted nucleic acid in an earlier study was divided into two aliquots, with one of the aliquots being subjected to DNA and the other to RNA processing for NGS, while in the current work each extracted nucleic acid was further treated by each procedure in duplicate.

The evaluation of viral nucleic acid extraction efficiency is different between the NGS and qRT-PCR analysis. The RPMK was most applicable for metagenomic analysis of viral RNA and enabled more sensitive identification of the RNA virus genome in respiratory clinical samples. Viral RNA extraction kits were also applicable for metagenomic analysis of the DNA virus. The results obtained in this study may differ if the NGS workflow and sequencing are not performed with the NEB Next® Ultra™ DNA library Prep Kit and Illumina Hiseq 2500 system. Further study will explore the influence of different extraction methods on the metagenomic analysis of viral nucleic acid using diverse biological samples including human feces, blood, and tissues containing multiple viral agents.

## Conclusions

The evaluation of viral nucleic acid extraction efficiency is different between NGS and qRT-PCR analysis. The RPMK was most applicable for the metagenomic analysis of viral RNA and enabled more sensitive identification of the RNA virus genome in respiratory clinical samples. In addition, viral RNA extraction kits were also applicable for metagenomic analysis of the DNA virus. Our results highlighted the importance of nucleic acid extraction kit selection, which has a major impact on the yield and number of viral reads by NGS analysis. Therefore, the choice of extraction method for a given viral pathogen needs to be carefully considered.

## Methods

### Clinical virus specimen

A spiked mixture was from two nasopharyngeal aspirate specimens, which contained ADV, Flu A, HPIV3, OC43, and HMPV. Before pooling, we used the cador Pathogen 96 QIAcube HT Kit (Qiagen) for automated viral DNA and RNA extraction with the QIAcube HT System. The presence of each virus was tested by individual qRT-PCR using specific primers and probes targeting different genomes [23]. The Ct values for the ADV, Flu A, PIV3, OC43, and HMPV were 23.9, 26.5, 17.1, 29.2, and 27.1, respectively. The Ct values are inverse to the nucleic acid concentration in correlation with the number of copies in the sample. Therefore, the lower the Ct values, the more abundant the nucleic acid presence. A 200-μL aliquot of the mixture was subjected to subsequent extraction in duplicate (*n* = 2) using four commercially available kits.

### Extraction kits

Four commercially available kits (VRMK, MVSK, RPMK, and RMK) were compared using simultaneous isolation of viral DNA and RNA, even though some kits were primarily designed exclusively for DNA or RNA (Table 5). The selection of the individual kits was based on their commercial availability and literature reports. The RPMK is used for purification of total RNA with the gDNA eliminator columns from small samples, including animal and human cells, tissues and microdissected cryosections, and for RNA cleanup and concentration. Major differences among the utilized commercial kits are their different chaotropic salts, detergents, and other additives included in the lysis buffers.

**Table 5:** Comparison of different extraction kits

Kit^a^	Target	Specimen type	Requirement		Starting volume^c^ (*μ*L)	Elution volume^c^ (*μ*L)
			Reagents^b^	special equipment		
VRMK	viral RNA	Plasma, serum and cell-free body fluids	–	–	200	50
MVSK	viral RNA, viral DNA	Plasma, serum and cell-free body fluids	–	–	200	50
RPMK	RNA	cells, tissues	β-mercaptoethanol	gDNA Eliminator spin column	200^d^	50^d^
RMK	viral RNA	cells, tissues	β-mercaptoethanol	–	200	50

^a^VRMK, QIAamp Viral RNA Mini Kit; MVSK, QIAamp MinElute Virus Spin Kit; RPMK, RNeasy Plus Micro Kit; RMK, RNeasy Mini Kit

^b^Not included in the kit

^c^In order to compare the four kits, starting and elution volume must be the same

^d^RNeasy Plus Micro Kit:maximum amount of starting material (animal and human cells): 5 × 10^5^, minimum elution volume: 14 μL

### Nucleic acid extraction

The mixed sample was homogenized by vortexing. Nucleic acid was extracted in parallel from 200 μL of the aliquot in duplicate for each kit, according to each of the manufacturer’s instructions (processed in the absence of viral enrichment). No carrier RNA was used for the extraction. Finally, the extracted nucleic acid was eluted individually (*n* = 8) in the same volume of 50 μL of the AVE buffer or RNase-free water.

### Molecular confirmation of viral infection

Following nucleic acid extraction by the four kits (Table 5), their individual performance with regard to the yield of viral nucleic acids was compared using qRT-PCR [23]. Specific qRT-PCR protocols were individually performed in duplicate (*n* = 16) for each virus and each extract (*n* = 8), using the 7500 Real Time PCR System (Applied Biosystems) for quantification of the ADV, OC43, Flu A, HMPV, and PIV3. The PCR mixtures consisted of 7.5 μL of the qRT-PCR buffer mix, 7.5 μL of each primer/probe set, and 5 μL of the 5 × enzyme mix (AgPath-ID™ One-Step RT-PCR Kit, Applied Biosystems). All qRT-PCR experiments were performed in a final 25-μL reaction volume containing 5 μL of the nucleic acid elute with the following cycling conditions: 30 min at 50 °C, 5 min at 95 °C, 40 cycles of 10 s at 95 °C, and 45 s at 55 °C.

### Quantification of total nucleic acid

Prior to further library processing, the yield of total extracted nucleic acid was quantified using the Qubit assay kit on the Qubit® 2.0 Flurometer. The Qubit® dsDNA HS Assay Kit (Invitrogen) is highly selective for double-stranded DNA and is designed to be accurate for the initial sample concentrations between 10 pg/μL and 100 ng/μL. The Qubit® RNA HS Assay Kit (Invitrogen) is designed to be accurate for RNA sample concentrations between 250 pg/μL and 100 ng/μL.

### Reverse transcription, library preparation and sequencing

Each viral extract (*n* = 8) was subjected to reverse transcription and PCR amplification. Eleven microliters of the elute were used as a template in a total volume of 20 μL, with 1 μL of random primer (50 μM), 1 μL of dNTPs (10 mM), 4 μL of 5 × first strand buffer, 1 μL of DTT (0.1 M), and 1 μL (200 units/μL) of SuperScript III (Invitrogen). The template and random primers were heated for 5 min at 65 °C, followed by reverse transcription for 60 min at 42 °C, and inactivation for 5 min at 96 °C. Prior to the second strand synthesis, cDNA was denatured for 2 min at 94 °C and cooled down for 5 min at 10 °C. The second strand was synthesized with 5 U/μL Klenow fragment exo-polymerase (Thermo Fisher Scientific) in a final volume of 10 μL and incubated at 37 °C for 30 min, followed by an enzyme inactivation step at 75 °C for 20 min. The resulting double-stranded cDNA and the originally extracted DNA fraction were further purified with the MinElute PCR Purification Kit (Qiagen).

Sequencing libraries were prepared with individual indices using the NEB Next® Ultra™ DNA library Prep Kit for Illumina (NEB, USA), following manufacturer’s recommendations. Index codes were added to attribute sequences to each sample. Briefly, the DNA sample was fragmented by sonication to a size of 300 bp, then DNA fragments were end-polished, A-tailed, and ligated with the full-length adaptor for the Illumina sequencing with further PCR amplification (8 cycles). Finally, the PCR products were purified (AMPure XP system) and libraries were analyzed for size distribution by the Agilent 2100 Bioanalyzer and quantified using RT-PCR. Sequencing was performed on the Illumina HiSeq 2500 system with the output of 2 × 150 bp paired-end reads. The clustering of the index-coded samples was performed on a cBot Cluster Generation System, according to the manufacturer’s instructions. After cluster generation, the library preparations were sequenced on the Illumina HiSeq2500 platform and paired-end reads were generated. The workflow was used to compare the performance of four different commercially available extraction kits on the selected viruses. The NGS runs (*n* = 8, corresponding to eight extractions) were performed in parallel.

### Bioinformatics analysis

The raw reads were filtered to remove low quality sequences and adapted with Trimmomatic (Version 0.36) and ng_QC (Version 1.0). After quality control was performed, the reads were further compared to the human reference genome hg19 and the aligned host reads were detected using the SoapAligner (Version 2.21). To assess the taxonomic assignment, the resulting reads for each sample were aligned with the virus database (July, 2015) and viral protein database from the NCBI Refseq database (July, 2015) using the VIP analysis software (Version 0.1.1) [24].

The sequences of five selected viruses (NCBI taxid 10,535, 162,387, 12,730, 11,216, 31,631, and 11,308) were extracted from the NCBI Refseq (August, 2017) and NCBI-NT (August, 2017) databases. In order to detect the selected viruses, all clean reads of each sample were mapped to the sub-Refseq (118 genomes) and sub-NT databases (396,146 sequences) with the SoapAligner (Version 2.21). Finally, for each sample, the reads that were mapped to the same species of the sub-NT database were assembled to contigs by the MEGAHIT (Version 1.1.1). The contigs were then mapped to the sub-NT database to determine the taxonomic classification.

## Additional file

Additional file 1: **Figure S1.** UPGMA (Unweighted Pair-group Method with Arithmetic Mean) analysis of the eight samples tested. Tree representing the results of the UPGMA hierarchical clustering of the weighted UniFrac distance matrix for each extraction in duplicates (*n* = 2) using four commercially available kits. The scale bar indicates the distance between clusters in UniFrac units. Four different extraction kits showed differences in grouping of eight extractions in Figure S1. The similarities and differences between the species and phylum communities in the four extraction kits were further quantified through UPGMA clustering analysis based on the weighted UniFrac distance metric. The results were clustered for each extraction kit and the parallel results within each extraction kit showed good repeatability. (DOCX 65 kb)

## Abbreviations

ADV: Adenovirus
bp: base pair
cDNA: complementary DNA
DNA: Deoxyribonucleic acid
dNTP: deoxynucleotide triphosphate
DTT: Dithiothreitol
Flu A: Influenzavirus A
HMPV: Human metapneumovirus
MVSK: QIAamp MinElute Virus Spin Kit
NGS: Next-generation sequencing
NT database: NCBI nucleotide collection database
nt: nucleotide
OC43: Coronavirus OC43
PIV3: Parainfluenza virus 3
qRT-PCR: quantitative reverse transcription polymerase chain reaction
RMK: RNeasy Mini Kit
RNA: Ribonucleic acid
RPMK: RNeasy Plus Micro Kit
SuperScript III: Superscript III reverse transcriptase
VRMK: QIAamp Viral RNA Mini Kit

## References

[1] Delwart EL (2007). Viral metagenomics. Rev Med Virol.

[2] Mokili JL, Rohwer F, Dutilh BE (2012). Metagenomics and future perspectives in virus discovery. Curr Opin Virol.

[3] Yang J, Yang F, Ren L, Xiong Z, Wu Z, Dong J, Sun L, Zhang T, Hu Y, Du J (2011). Unbiased parallel detection of viral pathogens in clinical samples by use of a metagenomic approach. J Clin Microbiol.

[4] Mokili JL, Dutilh BE, Lim YW, Schneider BS, Taylor T, Haynes MR, Metzgar D, Myers CA, Blair PJ, Nosrat B (2013). Identification of a novel human papillomavirus by metagenomic analysis of samples from patients with febrile respiratory illness. PLoS One.

[5] Lewandowska DW, Zagordi O, Geissberger FD, Kufner V, Schmutz S, Boni J, Metzner KJ, Trkola A, Huber M (2017). Optimization and validation of sample preparation for metagenomic sequencing of viruses in clinical samples. Microbiome.

[6] Li L, Deng X, Mee ET, Collot-Teixeira S, Anderson R, Schepelmann S, Minor PD, Delwart E (2015). Comparing viral metagenomics methods using a highly multiplexed human viral pathogens reagent. J Virol Methods.

[7] Hjelmso MH, Hellmer M, Fernandez-Cassi X, Timoneda N, Lukjancenko O, Seidel M, Elsasser D, Aarestrup FM, Lofstrom C, Bofill-Mas S (2017). Evaluation of methods for the concentration and extraction of viruses from sewage in the context of metagenomic sequencing. PLoS One.

[8] Wang C, Zhou S, Xue W, Shen L, Huang W, Zhang Y, Li X, Wang J, Zhang H, Ma X (2018). Comprehensive virome analysis reveals the complexity and diversity of the viral spectrum in pediatric patients diagnosed with severe and mild hand-foot-and-mouth disease. Virology.

[9] Klenner J, Kohl C, Dabrowski P, Nitsche A (2017). Comparing viral metagenomic extraction methods. Curr Issues Mol Biol.

[10] Zhou Y, Fernandez S, Yoon IK, Simasathien S, Watanaveeradej V, Yang Y, Marte-Salcedo OA, Shuck-Lee DJ, Thomas SJ, Hang J (2016). Metagenomics study of viral pathogens in undiagnosed respiratory specimens and identification of human enteroviruses at a Thailand hospital. Am J Trop Med Hyg.

[11] Gong YN, Yang SL, Chen GW, Chen YW, Huang YC, Ning HC, Tsao KC (2017). A metagenomics study for the identification of respiratory viruses in mixed clinical specimens: an application of the iterative mapping approach. Arch Virol.

[12] Schlaberg R, Queen K, Simmon K, Tardif K, Stockmann C, Flygare S, Kennedy B, Voelkerding K, Bramley A, Zhang J (2017). Viral pathogen detection by metagenomics and Pan-viral group polymerase chain reaction in children with pneumonia lacking identifiable etiology. J Infect Dis.

[13] Alquezar-Planas D, Mourier T, Bruhn C, Hansen A, Vitcetz S, Mørk S, Gorodkin J, Nielsen H, Guo Y, Sethuraman A (2013). Discovery of a divergent HPIV4 from respiratory secretions using second and third generation metagenomic sequencing. Sci Rep.

[14] Xu L, Zhu Y, Ren L, Xu B, Liu C, Xie Z, Shen K (2017). Characterization of the nasopharyngeal viral microbiome from children with community-acquired pneumonia but negative for Luminex xTAG respiratory viral panel assay detection. J Med Virol.

[15] Graf EH, Simmon KE, Tardif KD, Hymas W, Flygare S, Eilbeck K, Yandell M, Schlaberg R, Caliendo AM (2016). Unbiased detection of respiratory viruses by use of RNA sequencing-based metagenomics: a systematic comparison to a commercial PCR panel. J Clin Microbiol.

[16] Wang Y, Zhu N, Li Y, Lu R, Wang H, Liu G, Zou X, Xie Z, Tan W (2016). Metagenomic analysis of viral genetic diversity in respiratory samples from children with severe acute respiratory infection in China. Clin Microbiol Infect.

[17] Zou X, Tang G, Zhao X, Huang Y, Chen T, Lei M, Chen W, Yang L, Zhu W, Zhuang L (2017). Simultaneous virus identification and characterization of severe unexplained pneumonia cases using a metagenomics sequencing technique. Sci China Life Sci.

[18] Pei N, Zhang J, Ma J, Li L, Li M, Li J, Sun Y, Ji J, Jiang H, Hou Y (2016). First report of human salivirus/klassevirus in respiratory specimens of a child with fatal adenovirus infection. Virus Genes.

[19] Cheval J, Sauvage V, Frangeul L, Dacheux L, Guigon G, Dumey N, Pariente K, Rousseaux C, Dorange F, Berthet N (2011). Evaluation of high-throughput sequencing for identifying known and unknown viruses in biological samples. J Clin Microbiol.

[20] Rosseel T, Ozhelvaci O, Freimanis G, Van Borm S (2015). Evaluation of convenient pretreatment protocols for RNA virus metagenomics in serum and tissue samples. J Virol Methods.

[21] Chen Y, Trovão N, Wang G, Zhao W, He P, Zhou H, Mo Y, Wei Z, Ouyang K, Huang W, et al. Emergence and evolution of novel Reassortant influenza a viruses in canines in southern China. MBio. 2018;9(3). 10.1128/mBio.00909-18 PMC5989073 29871917

[22] Franzo G, Legnardi M, Hjulsager C, Klaumann F, Larsen L, Segales J, Drigo M (2018). Full-genome sequencing of porcine circovirus 3 field strains from Denmark, Italy and Spain demonstrates a high within-Europe genetic heterogeneity. Transbound Emerg Dis.

[23] Zhang D, Mao H, Lou X, Pan J, Yan H, Tang H, Shu Y, Zhao Y, Cheng X, Tao H (2018). Clinical evaluation of a panel of multiplex quantitative real-time reverse transcription polymerase chain reaction assays for the detection of 16 respiratory viruses associated with community-acquired pneumonia. Arch Virol.

[24] Li Y, Wang H, Nie K, Zhang C, Zhang Y, Wang J, Niu P, Ma X (2016). VIP: an integrated pipeline for metagenomics of virus identification and discovery. Sci Rep.

