# Supplementary material for: Metagenomic analysis of viral nucleic acid extraction methods in respiratory clinical samples
Source: BMC Genomics. 2018 Oct 25;19:773. doi: 10.1186/s12864-018-5152-5 (PMC6202819; doi:10.1186/s12864-018-5152-5)
Supplement: Supplementary file 1 — Figure S1. UPGMA (Unweighted Pair-group Method with Arithmetic Mean) analysis of the eight samples tested. Tree representing the results of the UPGMA hierarchical clustering of the weighted UniFrac distance matrix for each extraction in duplicates (n = 2) using four commercially available kits. The scale bar indicates the distance between clusters in UniFrac units. Four different extraction kits showed differences in grouping of eight extractions in Figure S1. The similarities and differences between the species and phylum communities in the four extraction kits were further quantified through UPGMA clustering analysis based on the weighted UniFrac distance metric. The results were clustered for each extraction kit and the parallel results within each extraction kit showed good repeatability. (DOCX 65 kb) [file 12864_2018_5152_MOESM1_ESM.docx]

Additional file 1


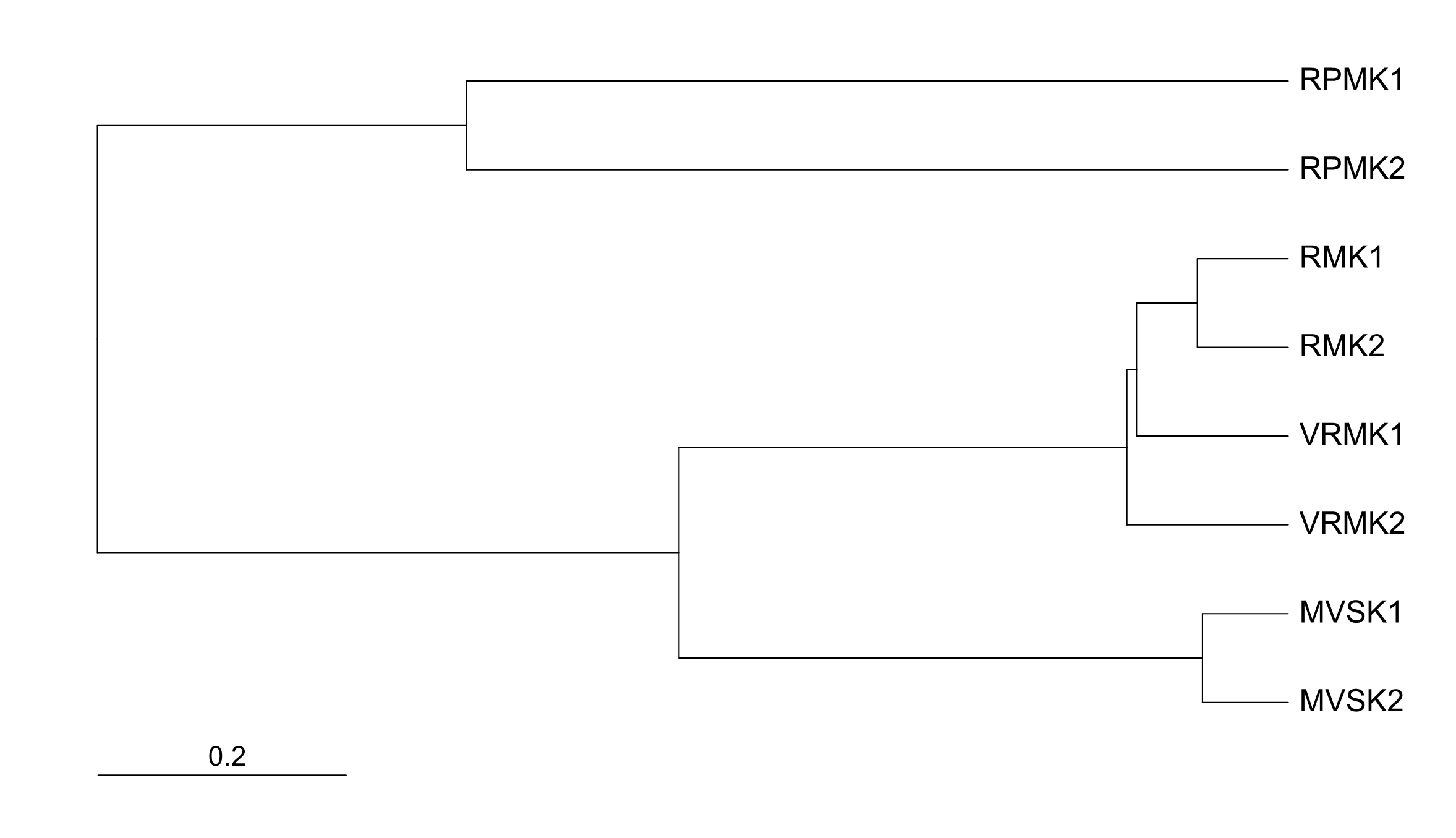


Figure S1 UPGMA(Unweighted Pair-group Method with Arithmetic Mean) analysis of the eight samples tested. Tree representing the results of the UPGMA hierarchical clustering of the weighted UniFrac distance matrix for each extraction in duplicates (n=2) using four commercially available kits. The scale bar indicates the distance between clusters in UniFrac units.

Four different extraction kits showed differences in grouping of eight extractions in Figure S1. The similarities and differences between the species and phylum communities in the four extraction kits were further quantified through UPGMA clustering analysis based on the weighted UniFrac distance metric. The results were clustered for each extraction kit and the parallel results within each extraction kit showed good repeatability.
